# MtABCG20 is an ABA exporter influencing root morphology and seed germination of *Medicago truncatula*

**DOI:** 10.1111/tpj.14234

**Published:** 2019-03-06

**Authors:** Aleksandra Pawela, Joanna Banasiak, Wanda Biała, Enrico Martinoia, Michał Jasiński

**Affiliations:** ^1^ Department of Plant Molecular Physiology Institute of Bioorganic Chemistry Polish Academy of Sciences Poznan Poland; ^2^ Department of Plant and Microbial Biology University of Zurich 8008 Zurich Switzerland; ^3^ Department of Biochemistry and Biotechnology Poznan University of Life Sciences Poznan Poland

**Keywords:** ABC transporters, abscisic acid, legumes, germination, root organ formation

## Abstract

Abscisic acid (ABA) integrates internal and external signals to coordinate plant development, growth and architecture. It plays a central role in stomatal closure, and prevents germination of freshly produced seeds and germination of non‐dormant seeds under unfavorable circumstances. Here, we describe a *Medicago truncatula* ATP‐binding cassette (ABC) transporter, MtABCG20, as an ABA exporter present in roots and germinating seeds. In seeds, *MtABCG20* was found in the hypocotyl–radicle transition zone of the embryonic axis. Seeds of *mtabcg20* plants were more sensitive to ABA upon germination, due to the fact that ABA translocation within *mtabcg20* embryos was impaired. Additionally, the *mtabcg20* produced fewer lateral roots and formed more nodules compared with wild‐type plants in conditions mimicking drought stress. Heterologous expression in *Arabidopsis thaliana* provided evidence that MtABCG20 is a plasma membrane protein that is likely to form homodimers. Moreover, export of ABA from *Nicotiana tabacum* BY2 cells expressing *MtABCG20* was faster than in the BY2 without *MtABCG20*. Our results have implications both in legume crop research and determination of the fundamental molecular processes involved in drought response and germination.

## Significance Statement

In the presented manuscript we introduce the MtABCG20, a half‐size ABC transporter of the G subfamily, as a modulator of abscisic acid distribution in model legume *Medicago truncatula*, participating in seed germination as well as root morphology and nodulation. Our results have implications both in legume crop research and determination of the fundamental molecular processes involved in drought response and germination.

## **Introduction**

Plant hormones (phytohormones) form a chemical communication system that integrates internal and external signals to coordinate plant development, growth and architecture. Abscisic acid (ABA) regulates plant water status, and promotes seed maturation and dormancy. Under drought stress conditions, ABA *inter alia* triggers stomatal closure, thus minimizing the loss of water through transpiration (Merilo *et al*., 2015), and changes hydraulic conductivity in a dose‐dependent manner (Dodd, 2013; Olaetxea *et al*., 2015) as well as modulates root system architecture to improve water uptake (Harris, 2015). ABA is also well‐recognized as a repressor of seed germination. It prevents germination of freshly produced seeds, and allows avoidance of germination in non‐dormant seeds under unfavorable circumstances (Chahtane *et al*., 2017). Additionally, in legume plants that establish a symbiotic interaction with nitrogen‐fixing bacteria, ABA controls, as a negative regulator, infection events in the epidermis and nodule primordium formation in the root cortex (Ding *et al*., 2008; Ding and Oldroyd, 2009).

Biosynthesis of ABA occurs predominantly in vascular cells, both in roots and shoots (Endo *et al*., 2008), as well as in the endosperm of seeds (Bethke *et al*., 2007). Some cell types expressing ABA receptors and perceiving the ABA signal are able to produce this hormone. The ABA synthesized *in situ* in guard cells is sufficient to trigger rapid stomatal closure in response to reduced air humidity (Bauer *et al*., 2013). However, the site of ABA biosynthesis is usually spatially separated from the site of action. Long‐term water deficiency engages ABA translocation through the vasculature (via the xylem), which must be later delivered toward guard cells by active transporters (Hu *et al*., 2016). Both the guard and vascular cells are functionally redundant in ABA production (Merilo *et al*., 2018).

Translocation of ABA within a plant occurs by passive diffusion, but is also mediated via primary and secondary transporters to ensure adequate response to environmental changes (Boursiac *et al*., 2013; Merilo *et al*., 2015). The following proteins that translocate ABA have been identified: (i) NRT1/PTR (NPF), originally recognized as nitrate or di/tri‐peptide transporters (Kanno *et al*., 2012); (ii) multidrug and toxic compound extrusion (MATE) proteins (Zhang *et al*., 2014), AWPM‐19 family protein member (Yao *et al*., 2018); and (iii) ATP‐binding cassette (ABC) transporters (Kuromori *et al*., 2010; Kang *et al*., 2015).

ABC transporters form one of the largest and most ancient protein superfamilies, with representatives in all extant phyla. They use ATP hydrolysis as a source of energy to transfer a wide variety of substrates through cellular membranes. In most cases, functional ABC transporters consist of two transmembrane domains (TMDs), which constitute the membrane‐spanning pore, and two cytosolic domains, which are referred to as the nucleotide‐binding domains (NBDs), as they contain the ATP‐binding Walker A and B motifs (Kang *et al*., 2011). Full‐size members are organized in a modular fashion, consisting of two pore‐forming TMDs alternating with two cytosolic NBDs. Half‐size members, which contain one TMD and one NBD, form homo‐ or hetero‐dimers that act as functional units. Based on structure and sequence homology, ABC proteins have been clustered into eight subfamilies (A–H; Verrier *et al*., 2008; Kang *et al*., 2011). Thus far, several members of the G subfamily, belonging to both the so‐called half‐size ABCG/WBC and full‐size ABCG/PDR proteins, have been reported as free‐ABA transporters (Borghi *et al*., 2015). Coordinated ABA translocation mediated by these transporters modulates physiological processes that are important for agricultural traits such as stomatal closure and seed germination (Kang *et al*., 2010, 2015; Kuromori *et al*., 2010, 2014, 2016).

Analyses of Arabidopsis mutants that exhibit an altered response to exogenously applied ABA led to the identification of two ABCG transporters involved in the intercellular movement of ABA. It was proposed that half‐size AtABCG25 (AtWBC26) acts as an exporter of ABA and releases this hormone from biosynthesizing cells (Kuromori *et al*., 2010). *AtABCG25* is co‐expressed in phloem companion cells with genes implicated in ABA biosynthesis (Kuromori *et al*., 2014). Transport experiments showed that AtABCG25 is a high‐affinity ABA efflux transporter (Kuromori *et al*., 2010). Finally, its overexpression in Arabidopsis resulted in reduced transpiration rates and consequently enhanced drought avoidance (Kuromori *et al*., 2016). Another research group discovered that full‐size AtABCG40 (AtPDR12) mediates specific ABA uptake into guard cells, where *AtABCG40* is strongly expressed and where intracellular ABA perception occurs. Phenotypic analyses dedicated to stomatal regulation revealed that *atabcg40* mutant lines exhibited higher transpiration rates and were more sensitive to desiccation. Moreover, tobacco and yeast cells overexpressing *AtABCG40* accumulated far more radiolabel‐ABA than control cells, whereas ABA influx into the *atabcg40* mutant protoplast was significantly reduced (Kang *et al*., 2010).

In addition to control of stomatal movement, it was reported that directional ABA transport mediated by ABCG transporters arrests germination. It was shown that four ABCG transporters localized in different seed tissues cooperate to repress seed germination by translocating ABA from the endosperm towards the target embryo. Half‐size AtABCG25 and full‐size AtABCG31 are involved in ABA efflux from the endosperm, where ABA is produced. ABA influx into embryo tissue is conducted by two importers, namely AtABCG30 and AtABCG40. A lack of these transporters resulted in disturbance of ABA distribution within seeds and shortened germination time in the case of knockout mutants, compared with the corresponding wild‐type (WT; Kang *et al*., 2015). Despite the described cooperative role of several ABCG transporters in ABA export from endosperm and import into the embryo, other transporters are likely to contribute to embryo fate. Recently, a spatially distributed signal and response system to ABA in dormant Arabidopsis seeds has been postulated to contribute to sensing and responding to external stimuli. It was proposed that increasing hormone transport rates within the embryo can further sensitize the system to change embryo fate in response to fluctuating external conditions (Topham *et al*., 2017). However, no such transporters have been identified, and the specific roles are yet to be established.

In contrast to Arabidopsis, the knowledge about ABA transporters in legumes is limited, although legumes are one of the main types of crops worldwide with important impact on farming, and also on animal and human nutrition. Moreover, numerous studies suggest a relevant role for this phytohormone and its distribution in legumes, for unique processes like nodulation (Suzuki *et al*., 2004; Ding *et al*., 2008; Tominaga *et al*., 2010). Here we present data demonstrating that MtABCG20 is an ABA transporter important for agricultural traits in legume crops.

## **Results**

### Gene expression pattern of *MtABCG20* in Medicago roots

**Figure 1:**
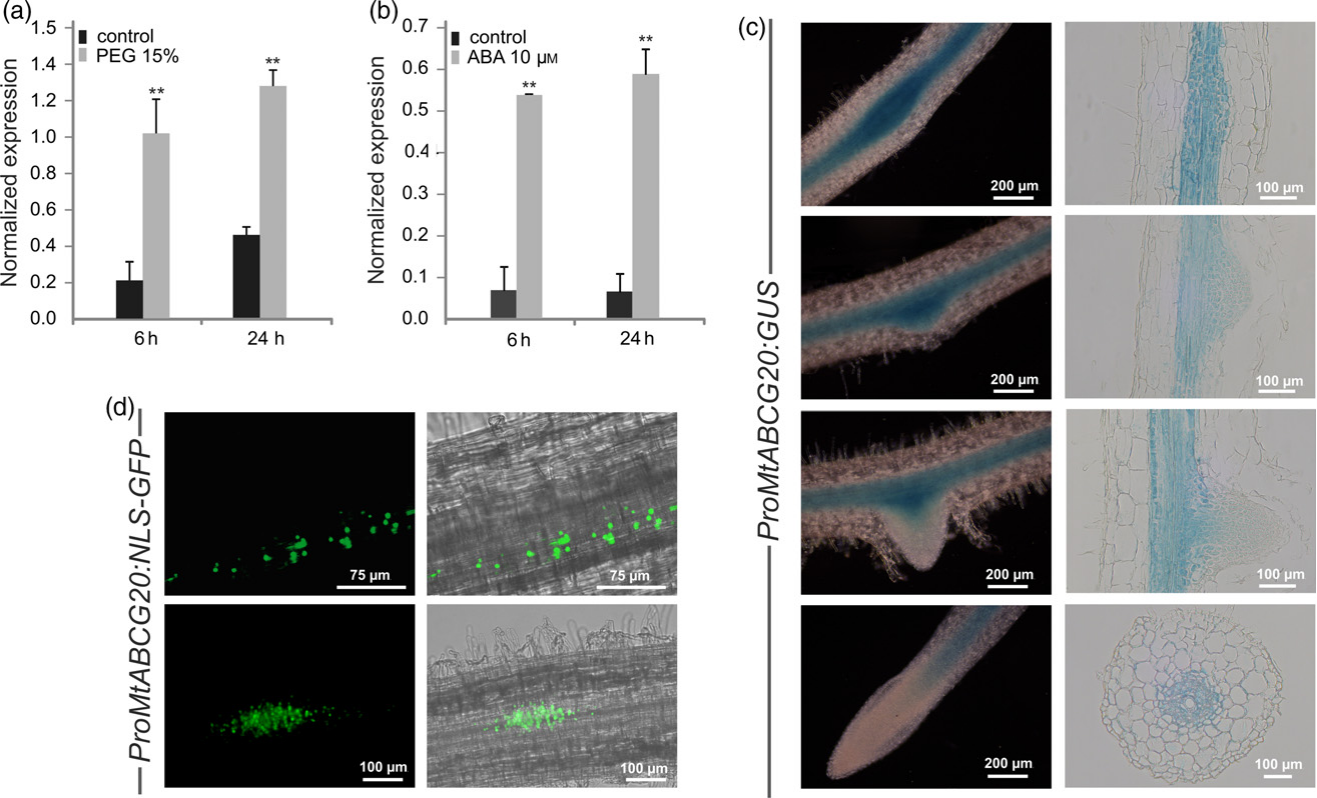
Expression analyses of *MtABCG20* in *Medicago truncatula* roots. Quantitative polymerase chain reaction (qPCR) time‐course expression analysis was performed for *MtABCG20* in roots treated with (a) 15% polyethylene glycol [PEG; real‐time (RT)‐PCR] or (b) 10 μm abscisic acid (ABA; Droplet Digital PCR). The transcript levels were normalized to the *Actin* gene. The data represent the mean ± SD of two independent biological experiments and three technical repeats. Significant differences from the control plants determined by Student's *t*‐test are indicated: **P < 0.01. (c) Promoter activity analyses of *MtABCG20* in transgenic *M. truncatula* roots. Expression of *ProMtABCG20:GUS* in the root (left panel) and root cross‐section (right panel). (d) Expression of *ProMtABCG20:NLS‐GFP*. Fluorescence images (left panel) and the merging of fluorescence and brightfield images (right panel).

Among 36 half‐size ABCG transporters identified in *Medicago truncatula* (Table S1), the mRNA of *MtABCG20* strongly accumulated 6 and 24 h after treatment of Medicago seedling roots with 15% polyethylene glycol (PEG; fold change > 2.5; Figure 1a) and 10 μm ABA (fold change > 6; Figure 1b), both mimicking drought stress conditions. To further investigate the *MtABCG20* expression profile, we generated *M. truncatula* composite plants expressing the β‐glucuronidase (GUS) reporter gene under the control of the native *MtABCG20* promoter (*ProMtABCG20:GUS*). Our analyses revealed a basal expression of *MtABCG20* along vascular bundles and at the sites of lateral root (LR) primordium formation (Figure 1c). The latter finding was additionally confirmed using *ProMtABCG20* fused with green fluorescent protein (GFP) containing a nuclear localization signal (NLS; Figure 1d; De Rybel *et al*., 2011). When *ProMtABCG20:GUS* transgenic Medicago hairy roots were challenged with 10 μm ABA, the expression pattern did not change in response to ABA, but an increased GUS signal intensity was observed (Figure S1). An expression pattern in the root vasculature similar to that of *MtABCG20* has been reported for genes encoding ABA biosynthesis enzymes (Koiwai *et al*., 2004; Endo *et al*., 2008), as well as for *AtABCG25* (Kuromori *et al*., 2010). AtABCG25 was previously described as a high‐affinity ABA exporter, and exhibits 47% identity at the protein level with MtABCG20. Interestingly, *MtABCG20* is also expressed in nodules (Figure S2).

### Root phenotype of the *MtABCG20* loss‐of‐function lines

**Figure 2:**
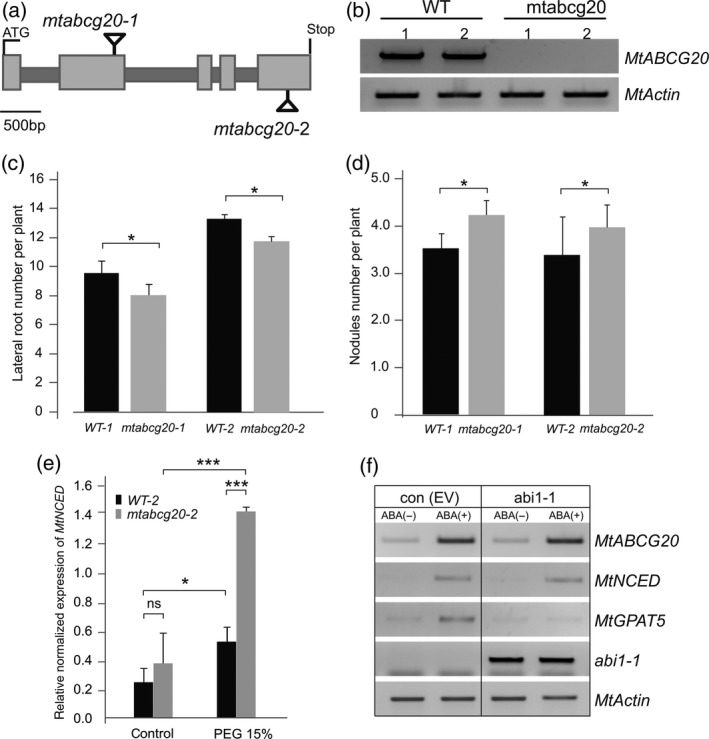
Phenotypic characterization of *mtabcg20* mutants. (a) Schematic diagram indicating Tnt1 insertions in two *mtabcg20* mutants, *mtabcg20‐1* (NF10694) and *mtabcg20‐2* (NF6539). Light gray and dark gray boxes indicate exons and introns of *MtABCG20*, respectively. (b) Full‐length *MtABCG20* mRNA in mutant lines analyzed by reverse transcription (RT)‐polymerase chain reaction (PCR). *Actin* used as an internal control. (c) Average lateral root (LR) number per plant in wild‐type (WT) and *mtabcg20* plants. All plants were grown for 4 weeks on ½ Murashige and Skoog (MS) medium containing 5% polyethylene glycol (PEG). Data represent the mean ± SD of three independent biological experiments on 30 plants (Student's *t*‐test *P < 0.05). (d) Average nodule number per plant in WT and *mtabcg20* plants. Three‐day‐old seedlings, pre‐treated with 10 μm abscisic acid (ABA), were inoculated with *Sinorhizobium meliloti* and grown on modified Fahraeus (‐N) medium. At 21 days post‐inoculation (dpi), nodule numbers were counted. The data represent the mean ± SD of two independent biological experiments with five technical repeats (eight plants each), per line (Student's *t*‐test *P < 0.05). (e) Real‐time PCR expression analyses of *MtNCED* in roots derived from WT‐2 and *mtabcg20‐2*, untreated or treated with 15% PEG. Transcript levels were normalized to the *Actin* gene. The data represent the mean ± SD of two independent biological experiments and three technical repeats. Significant differences between the groups were determined by Bonferroni *post hoc* tests following two‐way anova with the factors of genotype and condition: *P < 0.05, ***P < 0.001. (f) Semi‐quantitative PCR analyses of ABA‐dependent induction of *MtABCG20*,* MtNCED* and *MtGPAT5* in *M. truncatula* hairy‐root cultures transformed with empty vector (EV) or overexpressing *abi1‐1*, 24 h after 10 μm ABA treatment. Abi1‐1 primers were used to confirm *abi1‐1* allele expression in *M. truncatula* transgenic roots. The *Actin* transcript was used as an internal control.

Two tobacco retrotransposon (Tnt1) insertion lines have been identified for *MtABCG20*. One insertion is located in the second exon (NF10694, *mtabcg20‐1*) and another in the fifth exon (NF6539, *mtabcg20‐2*; Figure 2a). There was no detectable full‐length mRNA for *MtABCG20* in these two homozygous mutant lines, suggesting that *mtabcg20‐1* and *mtabcg20‐2* are null alleles (Figure 2b). Due to the fact that drought and ABA promotes LR formation (Gonzalez *et al*., 2015) and inhibits nodulation in Medicago (Ding *et al*., 2008), we sought to determine whether mutations in *MtABCG20* could affect root architecture in response to drought stress. To stimulate endogenous ABA production, 3‐day‐old WT and *mtabcg20* seedlings were transferred to medium containing 5% PEG. After incubation for 4 weeks, the number of LRs was counted, and statistically significant reduction in LR formation in *mtabcg20* was observed. The difference in LRs number between analyzed lines (NF10694 and NF6539) may result from distinct genetic backgrounds of them (Figure 2c). Moreover, the *mtabcg20* mutant produced approximately 18% more nodules than the WT during the drought stress mimicking (ABA pre‐treatment) condition (Figure 2d). The *mtabcg20* mutant and WT grown without PEG or ABA had no statistically significant differences in LR or nodule numbers, respectively (Figure S3).

Subsequently, we examined whether the disturbance in LR and nodules formation could be related to ABA arrest in biosynthesizing cells. For this reason, expression of *MtNCED* (9‐cis‐epoxycarotenoid dioxygenase) in WT and *mtabcg20* roots was compared. MtNCED is a key enzyme within the ABA biosynthetic pathway that is positively regulated by ABA at the transcript level (Sussmilch and McAdam, 2017). The quantitative polymerase chain reaction (qPCR) analyses showed that 24 h after PEG application the mRNA accumulation of *MtNCED* was in *mtabcg20* more than twice as high as in WT (Figure 2e). Additionally, the ABA‐dependent induction of *MtNCED* as well as *MtABCG20* was not affected in the Medicago lines overexpressing the Arabidopsis dominant‐negative allele of *abi1‐1*. The *abi1‐1* allele suppresses the ABA core signaling pathway in the effector cells where this phytohormone triggers responses to stresses (Wu *et al*., 2003). An example of such a response in roots is *inter alia* suberin production. In contrast to *MtNCED* and *MtABCG20*, the ABA‐dependent induction of *MtGPAT5*, encoding an acyltransferase engaged in suberin monomer biosynthesis (Beisson *et al*., 2007), was abolished in Medicago roots overexpressing *abi1‐1* (Figure 2f). The expression analyses that were performed indicate that lack of the MtABCG20 can possibly affect efflux of ABA from the biosynthesis place resulting in the observed *mtabcg20* root phenotypes.

### **Subcellular localization of MtABCG20**

**Figure 3:**
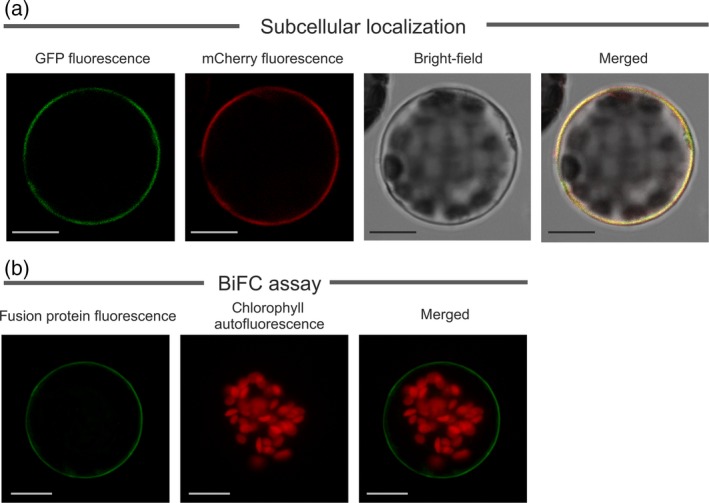
MtABCG20 plasma membrane localization and homodimer formation. (a) Co‐localization of fused green fluorescent protein (GFP)‐MtABCG20 and mCherry‐labeled plasma membrane marker AtPIP2A in Arabidopsis mesophyll protoplast. (b) Bimolecular fluorescent complementation (BiFC) assay demonstrating interaction of two MtABCG20 half‐size transporters. The fusion proteins Venus‐MtABCG20 and MtABCG20‐CFP were transiently expressed in Arabidopsis mesophyll protoplasts. Scale bar: 10 μm.

The subcellular localization of MtABCG20 was investigated *in planta* by transient expression of MtABCG20 fused N‐terminally to GFP under the control of the 35S Cauliflower Mosaic Virus (CaMV) promoter. Subcellular localization of the fusion protein was visualized by confocal microscopy imaging. The GFP signal was present around the cell and co‐localized with a plasma membrane marker, aquaporin AtPIP2A fused with mCherry (PM‐rK; Nelson *et al*., 2007; Figure 3a). As MtABCG20 is a half‐size ABCG protein, and it is known that half‐size ABCGs form dimers that act as functional transporters, we used the multicolor bimolecular fluorescent complementation (mcBiFC) assay to determine whether it dimerizes *in vivo*. Based on the modified pSAT series of vectors, two constructs were prepared: MtABCG20 with the C‐terminal portion of cyan fluorescent protein (cCFP); and MtABCG20 with the N‐terminal portion of Venus (nVenus). Arabidopsis leaf mesophyll protoplasts co‐transformed with cCFP‐MtABCG20, and nVenus‐MtABCG20 exhibited green fluorescence in the plasma membrane, indicating that MtABCG20 can form a homodimer (Figure 3b).

### **MtABCG20 is an abscisic acid exporter**

**Figure 4:**
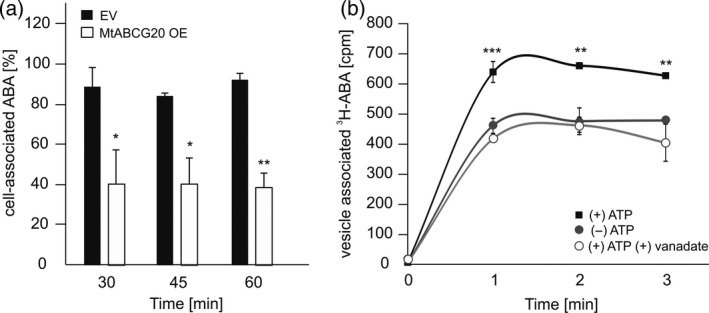
Abscisic acid (ABA) transport assays in BY2 cells and cell‐derived vesicles. (a) ABA efflux from BY2 control (EV) and *MtABCG20*‐overexpressing cell lines, conducted at 22°C and monitored by HPLC/MS. The 100% represents a quantity of cell‐associated ABA, defined as the ratio of the single‐ion chromatogram peak area to the internal standard, at time 0 (T0). Values represent the mean of three independent experiments ± SD. Significant differences between control and overexpressing lines determined by Student's *t*‐test are indicated: *P < 0.05, **P < 0.01. (b) Transport of ^3^H‐ABA into membrane vesicles derived from BY2 cells overexpressing *MtABCG20* in the absence of ATP as well as in the presence of ATP with or without orthovanadate. Values represent the mean of three independent experiments ± SD. Significant differences between ^3^H‐ABA uptake in the presence of ATP in comparison to other conditions were determined by an anova test and Tukey's multiple comparison test, and are as follows: **P < 0.01, ***P < 0.005.

To examine whether MtABCG20 can transport ABA through the plasma membrane, the *Pro35S:GFP‐MtABCG20* construct was heterologously expressed in *Nicotiana tabacum* BY2 cells. The presence of the protein of interest was confirmed by Western blotting using anti‐GFP antibodies (Figure S4), and its plasma membrane localization was determined by confocal microscopy (Figure S5). After preloading *MtABCG20*‐expressing or control cells (transformed with empty vector) with ABA, efflux of this phytohormone from BY2 cells was monitored using HPLC/MS. Deuterated ABA was used as an internal standard for HPLC/MS. ABA efflux from BY2 cells was significantly faster in cells transformed with *MtABCG20* compared with those transformed with the empty vector (Figures 4a and S6). The ABA transport is ATP‐dependent, as revealed by the assays conducted with radiolabeled ^3^H‐ABA and inside‐out membrane vesicles isolated from BY2 cells overexpressing *MtABCG20* (Figure 4b).

### **Expression pattern of** ***MtABCG20*** **in the seeds**

**Figure 5:**
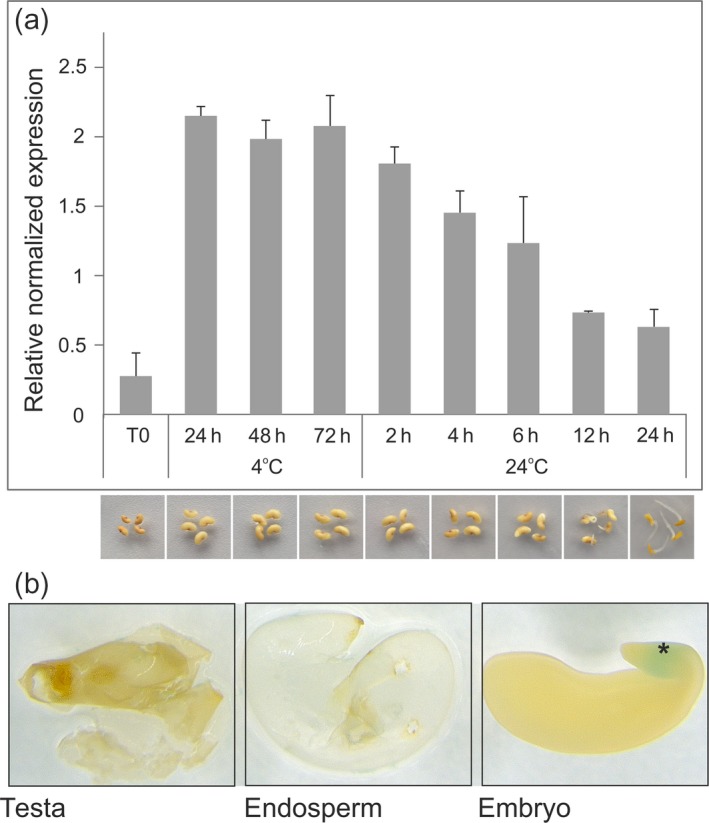
Expression analyses of *MtABCG20* in *Medicago truncatula* seeds. (a) Quantitative polymerase chain reaction (qPCR) time‐course expression analysis of *MtABCG20* during seed germination. Data represent the mean ± SD of two independent biological experiments and three technical repeats. Transcript levels were normalized to the *Actin* gene. Images below the graph show the Medicago seeds at different time points during germination. (b) *MtABCG20* promoter activity analyses in *M. truncatula* seeds using the β‐glucuronidase (GUS) reporter system. Seeds were stained for GUS activity and, consequently, particular seed parts (testa, endosperm, end embryo) were separated and visualized by light microscopy. *hypocotyl–radicle transition zone of embryo.

Abscisic acid participates not only in transmitting environmental stress signals such as drought but also in seed germination. It was recently shown that ABA transporters belonging to the ABCG subfamily influence seed germination in Arabidopsis (Kang *et al*., 2015). To explore whether MtABCG20 plays a role in seed biology, we analyzed the expression of the corresponding gene during seed germination. Scarified mature WT seeds were collected at various time points during germination progress. Quantitative real‐time (qRT)‐PCR analyses revealed that *MtABCG20* mRNA accumulated strongly after imbibitions, and remained relatively constant in seeds incubated at 4°C for the next 1, 2 and 3 days. After stratification, we observed a gradual decline of *MtABCG20* transcripts with the initiation of radicle emergence and germination completion (Figure 5a). To define the location of *MtABCG20* expression within the seed, we examined its promoter activity using the GUS reporter system, and observed that *MtABCG20* is expressed in the hypocotyl–radicle transition zone of the embryonic axis. *MtABCG20* promoter activity was detected neither in the endosperm layer nor testa (Figure 5b).

### **MtABCG20 plays a role in germination and mediates the export of abscisic acid from the hypocotyl–radicle zone**

**Figure 6:**
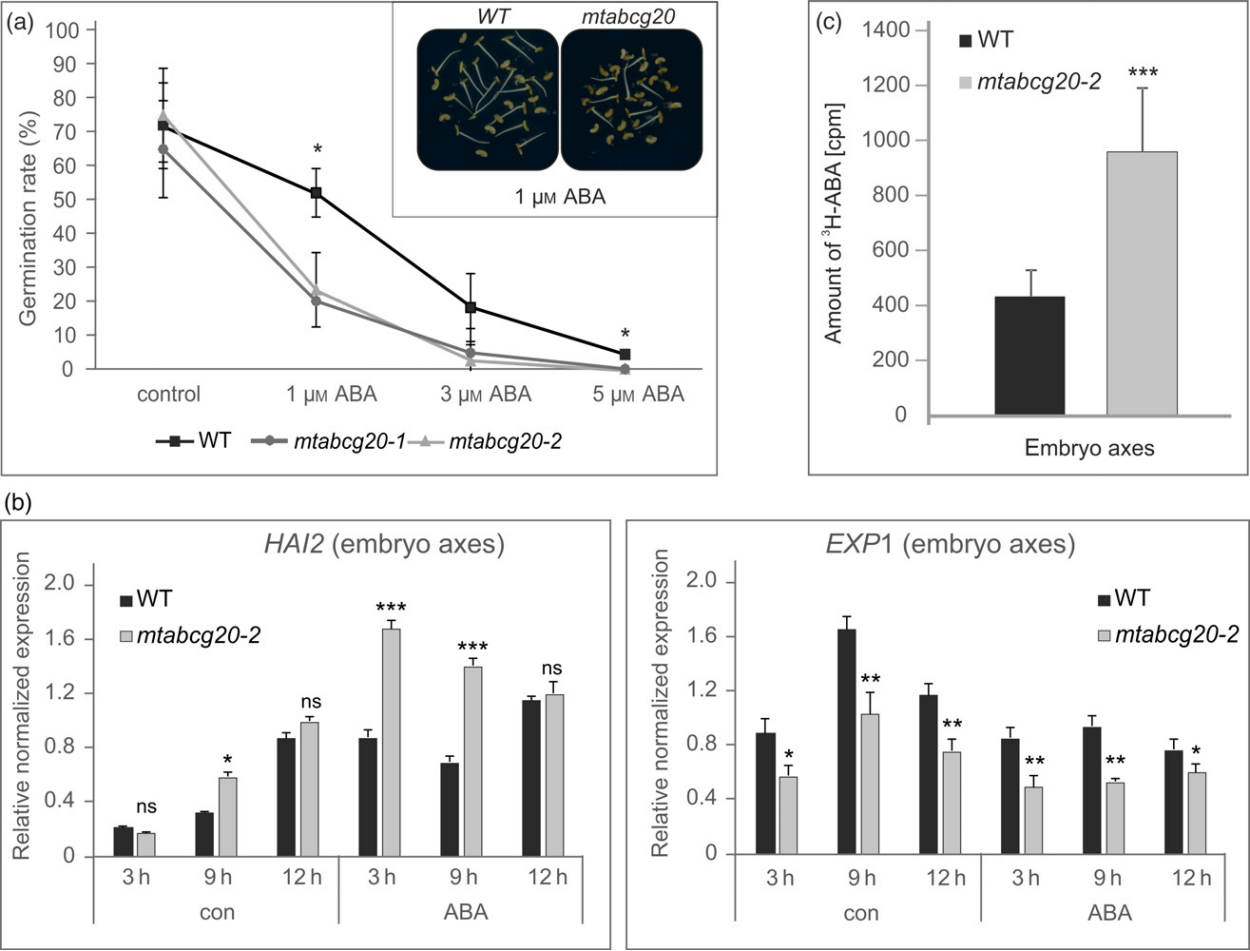
Seed germination phenotype of the *mtabcg20* mutant. (a) Germination assay of wild‐type (WT) and *mtabcg20* seeds. Stratified seeds were imbibed in the presence of different concentrations of abscisic acid (ABA) for 3 days at 4°C in the dark, and then moved to 23°C and scored 24 h after stratification. Each value represents the average percentage of germination of 30 seeds ± the SD of three replicates. Asterisks indicate significant differences of each *mtabcg20* line compared with WT based on Student's *t*‐test (*P < 0.05). (b) Real‐time polymerase chain reaction (PCR) expression analyses of *MtHAI2* and *MtEXP1* in embryo axes derived from WT and *mtabcg20* dissected embryos, untreated or treated with ABA applied onto the hypocotyl–radicle region. Transcript levels were normalized to the *Actin* gene. Results are presented as mean ± SD of three technical replicates of one representative biological repeat. Significant differences from the WT plants determined by Student's *t*‐test are indicated: *P < 0.05, **P < 0.01, ***P < 0.001. (c) Accumulation of ^3^H‐ABA in the embryo axis of WT and *mtabcg20* dissected embryos over 2 h; 1 μl of ^3^H‐ABA (0.15 mCi mmol^−1^) was applied to the embryo axis. Data are means ± SD of *n *= 30. Significant differences from the control (WT) determined by Student's *t*‐test are indicated: ***P < 0.001.

The germination assays using *mtabcg20* and WT seeds, which were stored for 1 month at room temperature and imbibed in the presence of different concentrations of ABA, revealed that *mtabcg20* seeds exhibit an enhanced sensitivity to ABA compared with the WT (Figure 6a).

Because cell wall loosening and cell expansion occur in the hypocotyl–radicle junction, leading to radicle emergence (Sliwinska *et al*., 2009), we hypothesized that MtABCG20 facilitates germination by extrusion of ABA from the hypocotyl–radicle transition zone. To verify our assumption, we monitored changes in the expression level of ABA‐responsive genes, which indirectly reflect ABA concentrations, in WT and *mtabcg20* mutant embryo axes (hypocotyl and radicle) and in cotyledons after application of ABA (Experimental scheme, Figure S7). For this purpose, embryos were isolated from scarified and stratified imbibed seeds. Consequently, the embryos were transferred onto microscope slides covered by solid medium supplied with 10 μm ABA, in such a way that only the embryo axes were in contact with the medium. Control embryos were placed similarly, but on medium without ABA. Next, embryo axes and cotyledons were collected 3, 9 and 12 h after ABA treatment, and used for RNA isolation. qRT‐PCR has been performed for the gene‐encoding components of the ABA signaling pathway (*HAI2*, highly ABA‐induced PP2C gene 2) and cell expansion during germination completion (*EXP1*, expansin A1‐like gene; Gimeno‐Gilles *et al*., 2009; Hyung *et al*., 2014; Maia *et al*., 2014). The analyses revealed that expression of *HAI2* was induced after ABA application in the embryo axes. Interestingly, in *mtabcg20*, the *HAI2* expression was higher in embryo axes compared with WT. In contrast, the expression of *EXP1* was downregulated by ABA, and its mRNA accumulation in the embryo axes was lower in *mtabcg20* than in WT (Figures 6b and S8). Concomitantly, in the cotyledons, the expression of *HAI2* was reduced in *mtabcg20* compared with WT (Figure S9). *EXP1* is not expressed in cotyledons in WT or *mtabcg20*. This result supports our assumption about the contribution of MtABCG20 to ABA extrusion from the embryonic axis to neighbor tissues. To further illustrate that ABA translocation from the hypocotyl–radicle transition zone takes place and is dependent on MtABCG20, we performed experiments with radiolabeled ABA. ^3^H‐ABA was applied to the embryonic axis of WT and *mtabcg20* dissected embryos, and its accumulation solely in the embryonic axis was monitored by scintillation counting (Figure S7). In the mutant line, we observed a higher level of radioactivity compared with the WT, suggesting that MtABCG20 dysfunction results in a disturbance of ABA removal from this region (Figure 6c).

## **Discussion**

Abscisic acid is a ubiquitous plant hormone, controlling plant growth and development as well as triggering responses to environmental stresses. Translocation of ABA within a plant is mediated by primary and secondary active transport systems that engage members from diverse protein families (Kang *et al*., 2010; Kuromori *et al*., 2010; Pellizzaro *et al*., 2014; Zhang *et al*., 2014). In this study, we characterized MtABCG20, an ABA transporter from *M. truncatula*. MtABCG20 is localized in the plasma membrane and is likely to form a functional homodimer, revealing ABA‐exporting activity. Expression of the corresponding gene has been detected in vascular parenchyma cells, where ABA biosynthesis mainly takes place. Its mRNA accumulates in roots after external addition of PEG and ABA.

**Figure 7:**
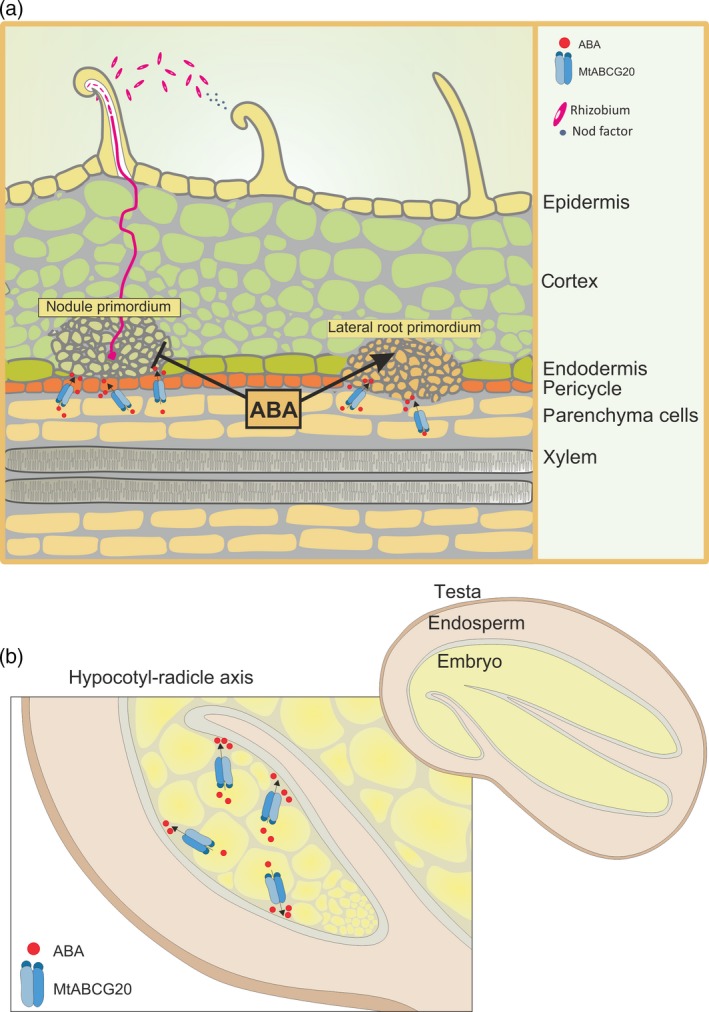
A proposed role of the MtABCG20 in Medicago roots and seeds.(a) MtABCG20 is an abscisic acid (ABA) exporter from biosynthesizing cells in roots (vascular parenchyma cells) enabling delivery of this hormone to the place where ABA‐dependent responses occur. In this way, MtABCG20 could positively affect lateral root (LR) primordium formation and exert a negative effect on the development of nodule primordia in Medicago. (b) In seeds MtABCG20 is responsible for extrusion of ABA from the hypocotyl–radicle transition zone, thereby facilitating germination.

There is a growing awareness of the role of ABA in the signaling of environmentally influenced plant developmental processes, *inter alia* in the modulation of the root architecture when a plant experiences abiotic stress (Harris, 2015). The role of ABA signaling in LR development is intricately connected to environmental responses and has different effects in different plant species. This is exemplified for instance by LR development in Arabidopsis and *M. truncatula*. While ABA signaling largely inhibits LR development in Arabidopsis (1 μm ABA is sufficient to block the development of visible LRs), ABA primarily plays a positive role in *M. truncatula* LR development, stimulating initiation, primordium formation, emergence and meristem activation at concentrations ranging from 0.1 to 10 μm (Gonzalez *et al*., 2015). It has been postulated that this altered ABA response has been acquired at the base of the legume lineage, coincident with the acquisition of a predisposition for nodulation (Liang and Harris, 2005). Notably, ABA transport likely functions in the regulation of LR meristem activation/formation, as the loss of the ABA importer in Arabidopsis, namely AtABCG40, results in a significant increase in LR formation in the presence of ABA (Kang *et al*., 2010). The role of ATP‐driven transporters in ABA signaling and root architecture is further strengthened by our observation that in Medicago, the ABA exporter MtABCG20 maintains LR number following osmotic stress. Considering the expression pattern of *MtABCG20* along the vascular bundles and at the LR primordium, it is likely that this transporter contributes to local changes in ABA concentration and, as a consequence, to LR formation. MtABCG20, similar to its homolog from Arabidopsis, ABCG25/WBC26, can be defined as a transporter responsible for the removal of ABA from its site of biosynthesis to the apoplast, enabling delivery of ABA to the place where ABA‐dependent responses occur (Figure 7a). This is supported by the observation that upon drought stress expression of *MtNCED*, positively regulated by ABA, in *mtabcg20* roots is stronger than in the WT. This might suggest that ABA is accumulated at its place of biosynthesis as a consequence of impaired export. The rather slight difference in LR number observed between WT and *mtabcg20* upon PEG treatment can be explained by ABA passive‐diffusion and/or the redundancy and functional compensation that often occurs among large protein families. Indeed, it was observed that mutants defective in ABA transport, belonging to ABCG subfamily (*atabcg25*,* atabcg40*), exhibit relatively mild phenotypes compared with typical ABA‐deficient mutants (Bhattacharjee *et al*., 2013; Danisman *et al*., 2013; Kuromori *et al*., 2018), and that several ABA transporters are involved in the import and export of ABA both in stomata and seeds (Kang *et al*., 2010, 2015; Kuromori *et al*., 2010, 2014).

In Medicago apart from *MtABCG20*, expression of another half‐size *ABCG* is upregulated in roots after ABA treatment, namely *MtABCG26* (Figure S10). The MtABCG26 is a homolog of AtABCG25, an ABA exporter from Arabidopsis (Figure S11). Further investigation is needed to demonstrate that MtABCG26 is an ABA transporter; however, the functional redundancy is possible. In legumes, LR formation and nodulation are adjusted by environmental inputs, hormone signaling, and signals exchanged between the root and rhizobia. ABA acts as a negative regulator for infection events in the epidermis and nodule primordium formation in the root cortex tissue (Ding *et al*., 2008; Ding and Oldroyd, 2009). The nodulation tests have shown that lack of MtABCG20 results in increased nodule number compared with WT. Increased nodule number can be also observed in Medicago after heterologous expression of the dominant‐negative allele of *abi1‐1* affecting the ABA core signaling pathway in the effector cells (Ding *et al*., 2008). However, such genetic inhibition of ABA signaling does not affect expression of neither *MtABCG20* nor *MtNCED*, suggesting that the nodule increase in *mtabcg20* is likely due to reduced export of ABA from its site of biosynthesis (Figure 7a). The significant, but rather small, effect may again be explained with redundancy of ABA transporters involved in this process.

Notably, in Medicago, other ABA transporters can also influence symbiotic nitrogen fixation. For instance, LATD/NIP, whose expression is regulated by ABA, is required for the establishment and maintenance of three root meristems, that of the primary root, LRs and symbiotic root nodules (Yendrek *et al*., 2010). Moreover, MtNPF6.8, a NRT1(PTR) family member shown to act as an ABA transporter, is involved in nitrate‐mediated inhibition of primary root growth that depends on ABA signaling (Pellizzaro *et al*., 2014). The effect of ABA is not limited to the early stages of nodule formation. It has been observed that the level of leghemoglobin that buffers free oxygen inside nodules largely declines after external addition of ABA (Gonzalez *et al*., 2001). The *Lotus japonicus* mutant known as enhanced nitrogen fixation 1 (*enf1*) accumulates lower amounts of endogenous ABA and exhibits higher activity of nitrogenase compared with the WT (Tominaga *et al*., 2010). *MtABCG20* is also expressed in the interior of the nodule and in vascular bundles developed at the nodule periphery. However, whether MtABCG20 only has an impact on determining nodule number or is also involved in the metabolic processes for nitrogen fixation must be further investigated.

It has been established that ABA plays a crucial role in embryo development, seed maturation, dormancy and germination. The latter starts with imbibition and completes with radicle emergence. The seed‐to‐seedlings transition represents a complex and critical developmental switch in the life‐cycle of higher plants, and its correct timing can determine seedling survival and subsequent reproduction (Shu *et al*., 2016). In Arabidopsis, four ABCG transporters can control seed germination through translocation of ABA from the endosperm into the embryo. Their coordinated action participates in embryo growth arrest and germination inhibition. Among them, AtABCG25 and AtABCG31 are localized in the endosperm and act as ABA exporters, while AtABCG30 and AtABCG40 are responsible for ABA uptake into the embryonic tissue (Kang *et al*., 2015). *MtABCG20* is also expressed in seeds, but its localization in the hypocotyl–radicle region of the embryonic axis attributes a new role to MtABCG20 (Sliwinska *et al*., 2009; Bassel *et al*., 2014). This region, which is well defined as a specific embryo growth zone, is the place where the elongation of embryo cells occurs to effect completion of germination (radicle emergence; Sliwinska *et al*., 2009). Interestingly, in *M. truncatula,* the inhibitory effect of ABA on germination/radicle emergence is associated with architectural modification, in this embryo axis, leading to the repression of cell wall loosening and cell expansion (Gimeno‐Gilles *et al*., 2009). However, the general mechanisms by which ABA inhibits seed germination, marked by the appearance of the radicle through the surrounding endosperm and testa, is still poorly understood. Several pieces of evidence support the hypothesis that ABA controls germination through the mobilization of reserves, but recent results favor an alternative hypothesis, namely that ABA acts through its direct effect on radicle emergence (Bethke and Jones, 2001; Gimeno‐Gilles *et al*., 2009). Taking into account the specific expression pattern of *MtABCG20* and the observation that *mtabcg20* mutants exhibited enhanced sensitivity to ABA during germination, we propose that MtABCG20 contributes to reducing the cellular ABA levels within the hypocotyl–radicle zone (Figure 7b). The latter activity, in addition to reduced uptake of ABA from the endosperm, changes the endogenous level of this hormone in the embryo. In agreement with this hypothesis is the observation that *mtabcg20* embryos are impaired in ABA translocation from the embryonic axes and hence remove ABA from the hypocotyl–radicle transition zone.

Notably, it was recently proposed that a spatial structure in germinating Arabidopsis seed/embryo may filter out noisy inputs from the environment and stimulate the termination of dormancy. In Arabidopsis embryos, the responses to ABA and gibberellin were found to occur within distinct cell types, suggesting communication between various signaling centers via hormone transport. The spatial separation of such signaling centers is required to process variable inputs from the environment and to promote the breaking of dormancy (Topham *et al*., 2017). ABA translocation within the embryo and the result of such signal transduction between biological compartments/cell types underlines the crucial role of transporters and the transport rate as decisive for the developing embryo and developmental fate. Notably, together with the activity of this transporter in environmentally regulated plant developmental processes, like the root architecture it can be postulated that the MtABCG20 comprehensively participates in response to environmental cues by modulating ABA transport. Its activity can be recognized as a regulatory element of seed germination as well as root morphology and nodulation.

## **Experimental Procedures**

### **Plant material and growth conditions**

*Medicago truncatula* (ecotype R‐108) seeds of the *mtabcg20* Tnt1 insertion mutant lines (NF10694, NF6539) were obtained from the Noble Research Institute.

*Medicago truncatula* (WT and mutants) seeds were chemically scarified with concentrated sulfuric acid, stratified (4°C in dark for 3 days), and transferred to ½ Murashige and Skoog (MS) agar medium. Seedlings were grown under controlled greenhouse conditions with a mean temperature of 22°C, 50% humidity and a 16‐h photoperiod.

*Medicago truncatula* hairy‐root cultures (control and overexpressed *abi1‐1*) were initiated from the 10‐mm root fragments containing meristem and growing them in the dark at 22°C. Cultures were grown on solid Fahraeus medium, supplemented with sucrose (10 g L^−1^), myoinositol (100 mg L^−1^), thiamine (10 mg L^−1^), pyridoxine (1 mg L^−1^), biotin (1 mg L^−1^), nicotinic acid (1 mg L^−1^) and glycine (2 mg L^−1^). Fragments of hairy roots were transferred onto fresh medium every 3 weeks.

*Nicotiana tabacum* Bright Yellow 2 (BY2) suspension cell cultures (Nagata *et al*., 1992) were grown in a MS basal salt mixture supplemented with 30 g L^−1^ sucrose, 0.2 mg L^−1^ 2,4‐dichlorophenoxyacetic acid, 1 mg L^−1^ thiamine, 100 mg L^−1^ myo‐inositol and 370 mg L^−1^ KH_2_PO_4_, in the dark at 26°C on an orbital shaker (130 rpm), and diluted 1:59 every 2 weeks.

### **Genetic constructs**

The promoter region of *MtABCG20* (1281 bp) was amplified and cloned into the following binary vectors: (i) pPR97, carrying the β‐glucuronidase (gusA) reporter gene (Szabados *et al*., 1995), by restriction/ligation using restriction sites for BamHI and EcoRI; and (ii) pPLV04_v2, carrying a GFP reporter gene tagged with a NLS, by ligation‐independent cloning (De Rybel *et al*., 2011). The cDNA fragment (2049 bp) corresponding to the CDS of *MtABCG20* used for overexpression and subcellular localization was amplified and cloned into pMDC43, carrying GFP (Curtis and Grossniklaus, 2003), by restriction/ligation using restriction sites for AscI and PstI. For the BiFC assay, the CDS of *MtABCG20* was cloned into pDONR™/Zeo (Invitrogen, Carlsbed, CA, USA) by site‐specific recombination using Gateway BP Clonase II Enzyme Mix (Invitrogen) and then recombined into the pSAT3‐nVenus‐DEST and pSAT5‐DEST‐cCFP vectors (Mitula *et al*., 2015) by site‐specific recombination using Gateway LR Clonase II Enzyme Mix (Invitrogen). For primer sequences, see Table S2.

### **Plant transformation**

Transgenic roots carrying *ProMtABCG20:GUS* or *ProMtABCG20:NLS‐GFP* constructs or *Arabidopsis abi1‐1 allele* were obtained from *M. truncatula* after infection of a radicle with *Agrobacterium rhizogenes* Arqua1. Stably transformed *M. truncatula* plants carrying *ProMtABCG20:GUS* were obtained by *Agrobacterium tumefaciens* AGL1‐mediated transformation using leaf explants and regeneration via somatic embryogenesis (http://www.noble.org/medicagohandbook). Stably transformed BY2 cells were generated by co‐cultivation with *A. tumefaciens* AGL1 carrying *Pro35S:GFP‐MtABCG20* or *Pro35S:GFP*, as previously described (Biala *et al*., 2017).

### **Abscisic acid and polyethylene glycol treatment**

Seven‐day‐old Medicago seedlings were transferred to solid ½ MS medium supplemented with 10 μm ABA or Whatman paper saturated with 15% PEG (PEG 6000 Serva 33137; osmotic potential −0.54 MPa) solution. Samples were collected at different time points (3, 6 and 24 h after transfer) and immediately frozen. The collected material was used for qRT‐PCR analyses.

### Quantitative real‐time‐polymerase chain reaction analyses

RNA was isolated from plant material with an RNeasy Extraction kit. Genomic DNA was removed by on‐column DNase treatment. Total RNA (500 ng) was converted to cDNA with Omniscript reverse transcriptase (Qiagen, Hilden, Germany) following the manufacturer's protocol. Droplet digital PCR was performed with the QX200 Droplet Digital PCR (ddPCR™) System (Bio‐Rad, Hercules, CA, USA) using EvaGreen. RT‐PCR was performed in a CFX Connect Real‐Time System machine (Bio‐Rad) using SYBR Green. *Actin* was used as a reference gene for normalization, and the gene expression levels were determined by the ΔΔCt method. For primer sequences, see Table S2.

### **Transport assays in suspension cell cultures**

Four‐day‐old suspension cell cultures (overexpressing *MtABCG20* or transformed with EV) were filtered, washed, and suspended in fresh, ice‐cold growth medium. After the addition of ABA (250 μm) as a substrate, the cells were incubated for 30 min at 4°C with agitation (60 rpm). After incubation, the cells were filtered, washed and transferred to fresh, growth medium (T0), and then incubated with agitation (60 rpm) at 22°C/18°C. Samples (5 ml of cell culture) were collected at the defined time points, filtered and frozen. Frozen cells were ground at 4°C with mortar and pestle, and extracted with 3 ml of 80% methanol. Dried extracted samples were dissolved in 200 μl of 80% methanol and analyzed by liquid chromatography–electrospray ionization–tandem mass spectrometry (LC/ESI/MS) using a Waters UPLC connected to a Bruker micrOTOF‐Q mass spectrometer (Staszkow *et al*., 2011). Deuterated ABA was used as the internal standard.

## **Accession numbers**

Sequence data from this article can be found in the GenBank database under the following accession numbers: MTR_1g093990 for MtABCG20, MTR_3g095530 for Actin, MTR_7g111010 for MtEXP1, MTR_5g080680 for MtHAI2, MTR_2g070460 for MtNCED, MTR_5g080360 for MtGPAT5.

## **Authors' contributions**

M.J. designed the research and interpreted results; A.P. and J.B. performed the majority of the experiments, contributed to experimental design and result interpretation; A.P. and W.B performed transport experiments; A.P. performed BiFC and embryo‐based experiments; J.B. conducted phenotypic characterization; M.J., J.B. and A.P. wrote the manuscript; E.M. contributed to experimental design and writing.

## **Conflict of interest**

The authors declare no conflict of interest.

## Supporting information


**Figure S1.** Promoter activity analyses of *MtABCG20* in transgenic *M. truncatula* roots.
**Figure S2.** Promoter activity analyses of *MtABCG20* in transgenic *M. truncatula* nodule.
**Figure S3.** Phenotypic characterization of *mtabcg20* mutants.
**Figure S4.** Expression of MtABCG20 in *N. tabacum* BY2 cells.
**Figure S5.** Plasma membrane localization of MtABCG20 in BY2 cells.
**Figure S6.** ABA transport assay in BY2 cells.
**Figure S7.** Experimental scheme of ABA application onto Medicago embryo.
**Figure S8.** Real‐time PCR expression analyses of *MtHAI2* and *MtEXP1* in embryo axes.
**Figure S9.** Real‐time PCR expression analyses of *MtHAI2* in cotyledons.
**Figure S10.** Changes of the selected (clustering with AtABCG25) half‐size *MtABCGs* expression in roots after exogenous ABA application.
**Figure S11.** Phylogenetic tree of half‐size ABCG proteins from *Arabidopsis thaliana* and *Medicago truncatula*.

**Table S1.** Accession numbers of *Medicago truncatula* half‐size ABCG genes (WBC).
**Table S2.** List of primers used in this study.
