# Supplementary material for: MtABCG20 is an ABA exporter influencing root morphology and seed germination of Medicago truncatula
Source: Plant J. 2019 Mar 6;98(3):511–23. doi: 10.1111/tpj.14234 (PMC6850635; doi:10.1111/tpj.14234)
Supplement: Supplementary file 1 — Figure S1. Promoter activity analyses of MtABCG20 in transgenic M. truncatula roots. Figure S2. Promoter activity analyses of MtABCG20 in transgenic M. truncatula nodule. Figure S3. Phenotypic characterization of mtabcg20 mutants. Figure S4. Expression of MtABCG20 in N. tabacum BY2 cells. Figure S5. Plasma membrane localization of MtABCG20 in BY2 cells. Figure S6. ABA transport assay in BY2 cells. Figure S7. Experimental scheme of ABA application onto Medicago embryo. Figure S8. Real‐time PCR expression analyses of MtHAI2 and MtEXP1 in embryo axes. Figure S9. Real‐time PCR expression analyses of MtHAI2 in cotyledons. Figure S10. Changes of the selected (clustering with AtABCG25) half‐size MtABCGs expression in roots after exogenous ABA application. Figure S11. Phylogenetic tree of half‐size ABCG proteins from Arabidopsis thaliana and Medicago truncatula. [file TPJ-98-511-s001.pdf]

SUPPORTING INFORMATION

Figure S1. Promoter activity analyses of *MtABCG20* in transgenic *M. truncatula* roots.

Figure S2. Promoter activity analyses of *MtABCG20* in transgenic *M. truncatula* nodule.

Figure S3. Phenotypic characterization of *mtabcg20* mutants.

Figure S4. Expression of MtABCG20 in *N. tabacum* BY-2 cells.

Figure S5. Plasma membrane localization of MtABCG20 in BY2 cells.

Figure S6. ABA transport assay in BY2 cells.

Figure S7. Experimental scheme of ABA application onto Medicago embryo.

Figure S8. Real-time PCR expression analyses of *MtHAI2* and *MtEXP1* in embryo axes.

Figure S9. Real-time PCR expression analyses of *MtHAI2* in cotyledons.

Figure S10. Changes of the selected (clustering with AtABCG25) half-size *MtABCGs* expression in roots after exogenous ABA application.

Figure S11. Phylogenetic tree of half-size ABCG proteins from *Arabidopsis thaliana* and *Medicago truncatula*.

Table S1. Accession numbers of *Medicago truncatula* half-size ABCG genes (WBC).

Table S2. List of primers used in this study.

Supporting Figures

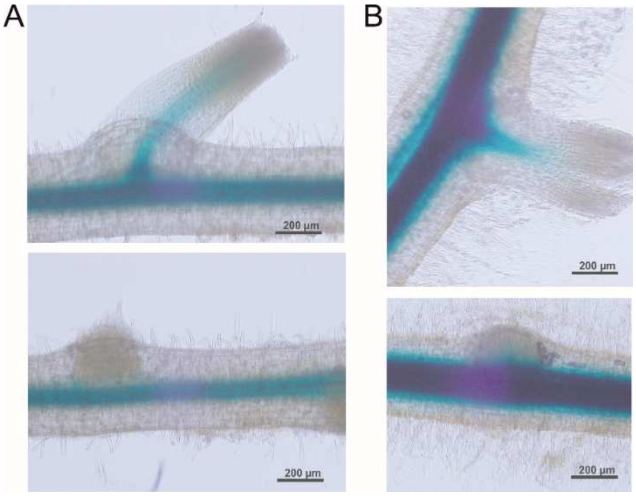

Figure S1. Promoter activity analyses of *MtABCG20* in transgenic *M. truncatula* roots. (A) Control, untreated transgenic roots. (B) Transgenic roots treated by 10 µM ABA.

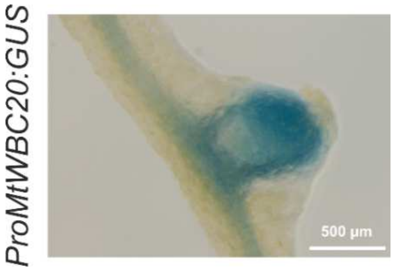

Figure S2. Promoter activity analysis of *MtABCG20* in transgenic *M. truncatula* nodule.

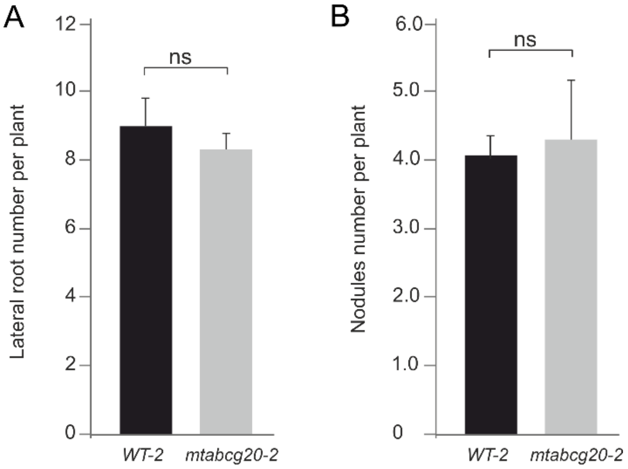

Figure S3. Phenotypic characterization of *mtabcg20* mutants. (A) Average lateral root number per plant in WT and *mtabcg20* plants. All plants were grown for four weeks on ½ MS medium. Data represent the mean ± SD of N=5, n=5. (B) Average nodule number per plant in WT and *mtabcg20* plants. 3-day-old seedlings, were inoculated with *S. meliloti* and grown on modified Fahraeus (-N) medium. At 21 days post-inoculation (dpi), nodule numbers were counted. The data represent the mean ± SD of N=5, n=5.

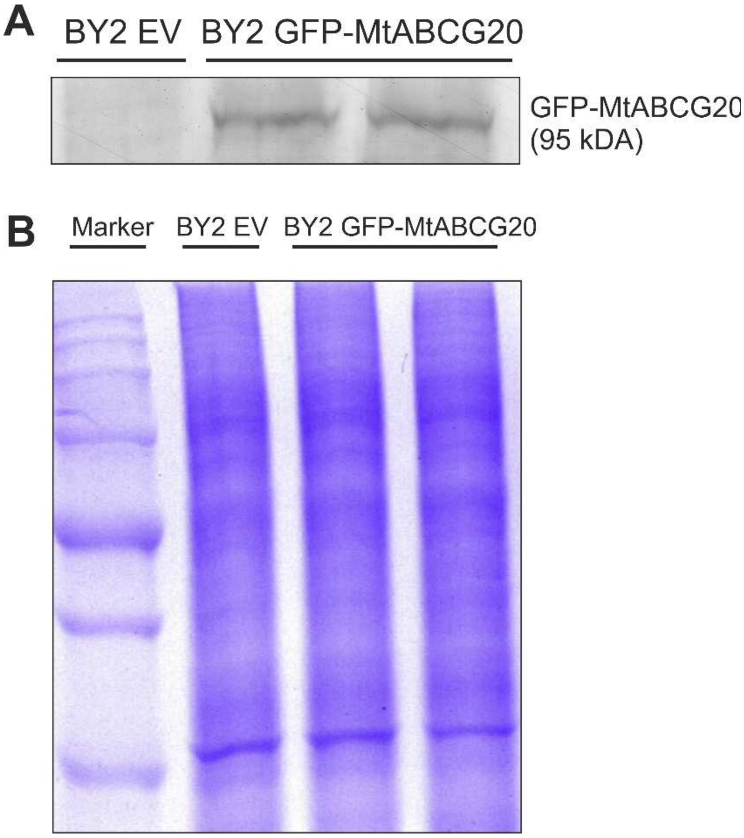

Figure S4. Expression of MtABCG20 in *N. tabacum* BY-2 cells. (A) Western blot analysis of the crude membranes (30 µg), obtained from BY2 transformed with empty vector (EV) and BY2 MtABCG20-overexpressing lines. Microsomal fractions were isolated as previously described (Jasinski et al., 2001) from 300 mg of BY2 cells. The proteins were separated by SDS-PAGE and transferred to a polyvinylidene fluoride membrane (Millipore) by electroblotting (semi-dry; apparatus; Bio-Rad). The membrane was incubated with a primary Anti-GFP from mouse (Roche) diluted 1/1000 and the secondary alkaline phosphatase-conjugated goat anti-mouse IgG (Abcam) diluted 1/15000. (B) Coomassie blue-stained gel with crude membrane fraction as a loading control. M - Perfect^{TM} Tricolor Protein Ladder (EURx).

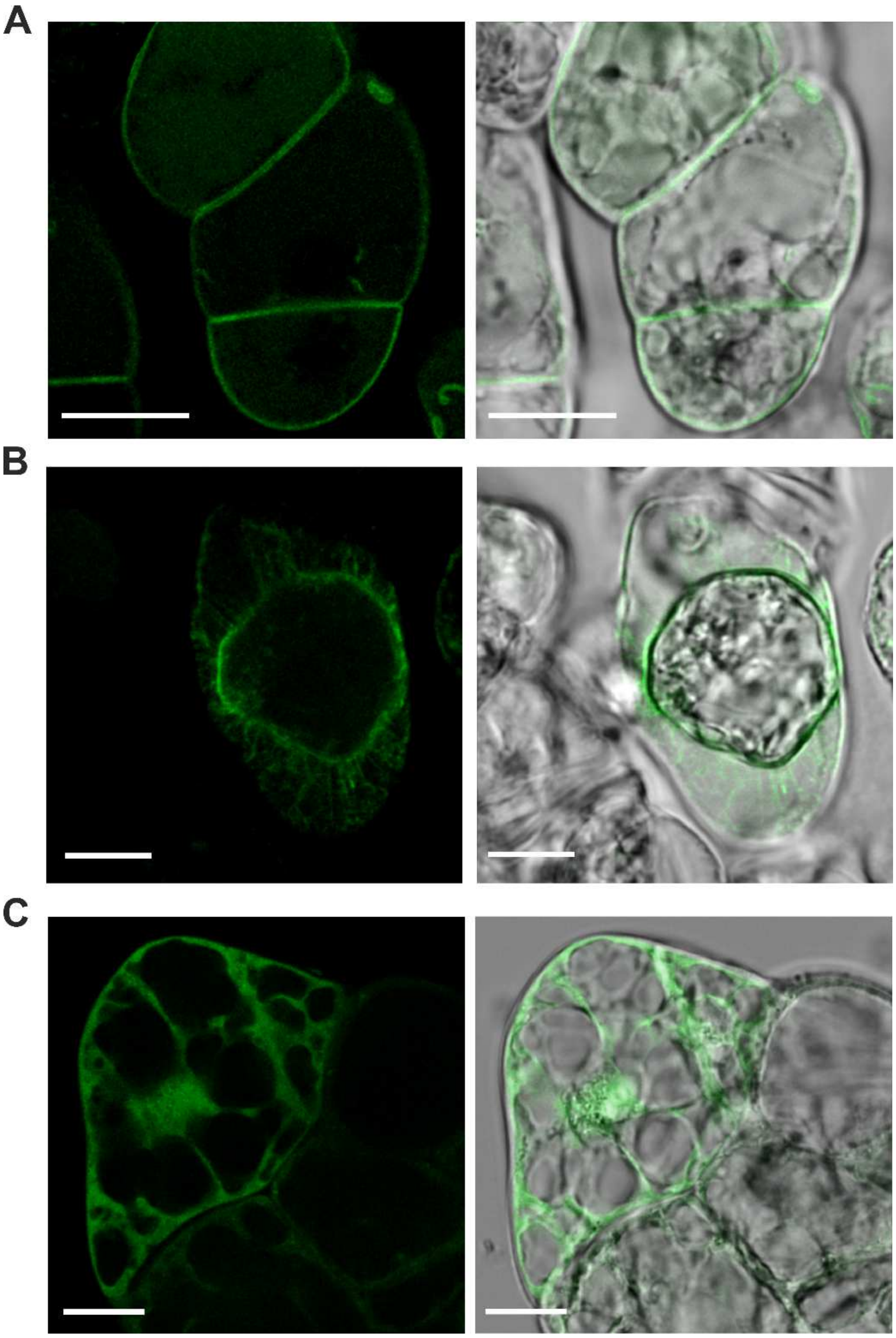

Figure S5. Plasma membrane localization of MtABCG20 in BY2 cells. (A) Non-plasmolysed BY2 cell expressing the fusion protein GFP-MtABCG20. GFP signal was localized on the surface of cells (B) Plasmolysed (5% NaCl for 10 min) BY-2 cells expressing the fusion protein GFP-MtABCG20. GFP signal was distributed on a plasma membranes and Hechtian strands. (C) Control BY2 cell expressing free cytoplasmic GFP. Bars = 15 μm.

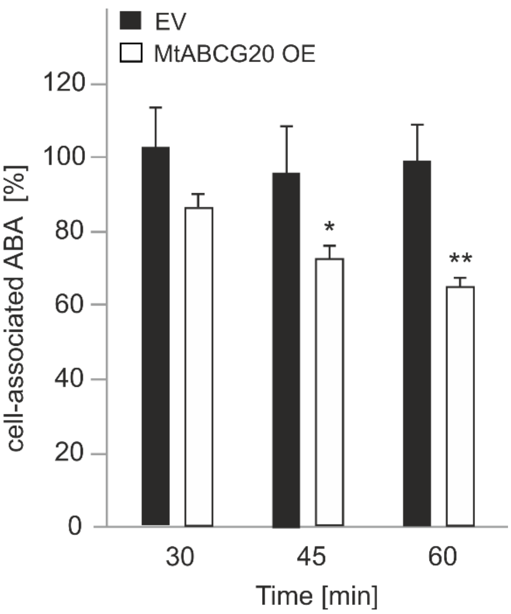

Figure S6. ABA transport assay in BY2 cells. ABA efflux from BY2 control (EV) and *MtABCG20*-overexpressing cell lines, conducted at 18°C and monitored by HPLC/MS. The 100% value represents the quantity of cell-associated ABA, defined as the ratio of the single-ion chromatogram peak area to the internal standard, at the time 0 (T0). Values represent the mean of three experiments ± SD. Significant differences between control and overexpressing lines determined by Student's t-test are indicated: *P < 0.05, **P < 0.01.

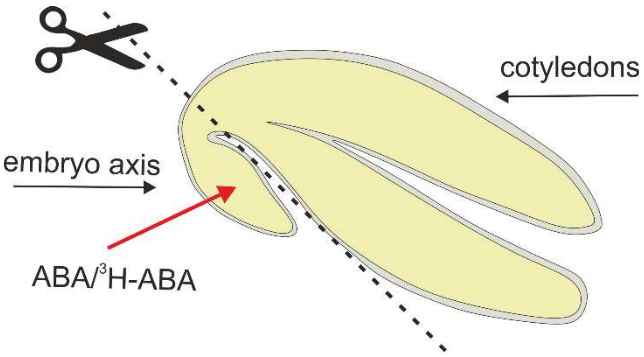

Figure S7. Experimental scheme of ABA application onto Medicago embryo**.**

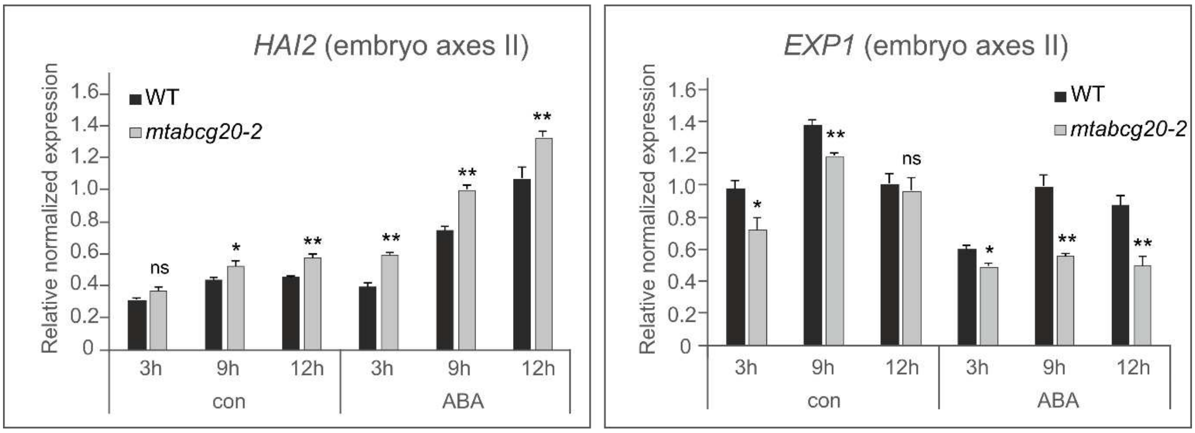

Figure S8. The second biological replicate of real-time PCR expression analyses of *MtHAI2* and *MtEXP1* in embryo axes derived from WT and *mtwbc20* dissected embryos. Embryos were untreated or treated with ABA applied onto the hypocotyl-radicle region. Transcript levels were normalized to the *Actin* gene. The data represent the mean ± SD of three technical repeats. Significant differences from the WT plants determined by Student's t-test are indicated: *P<0.05, **P < 0.01.

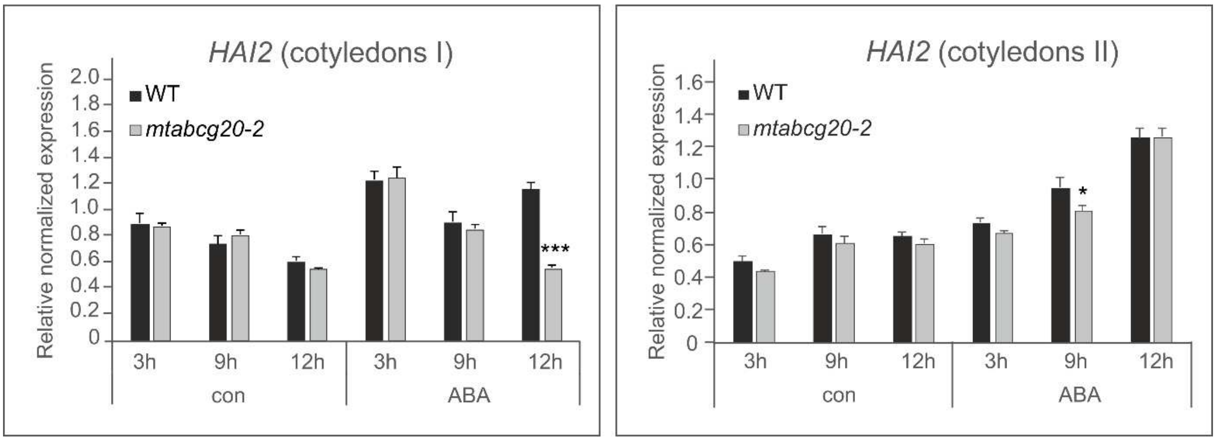

Figure S9. Two biological replicates of real-time PCR expression analyses of *MtHAI2* in cotyledons derived from WT and *mtwbc20* dissected embryos. Embryos were untreated or treated with ABA applied to the hypocotyl-radicle region. Transcript levels were normalized to the *Actin* gene. The data represent the mean ± SD of three technical repeats. Significant differences from the WT plants determined by Student's t-test are indicated: *P<0.05, ***P<0.001.

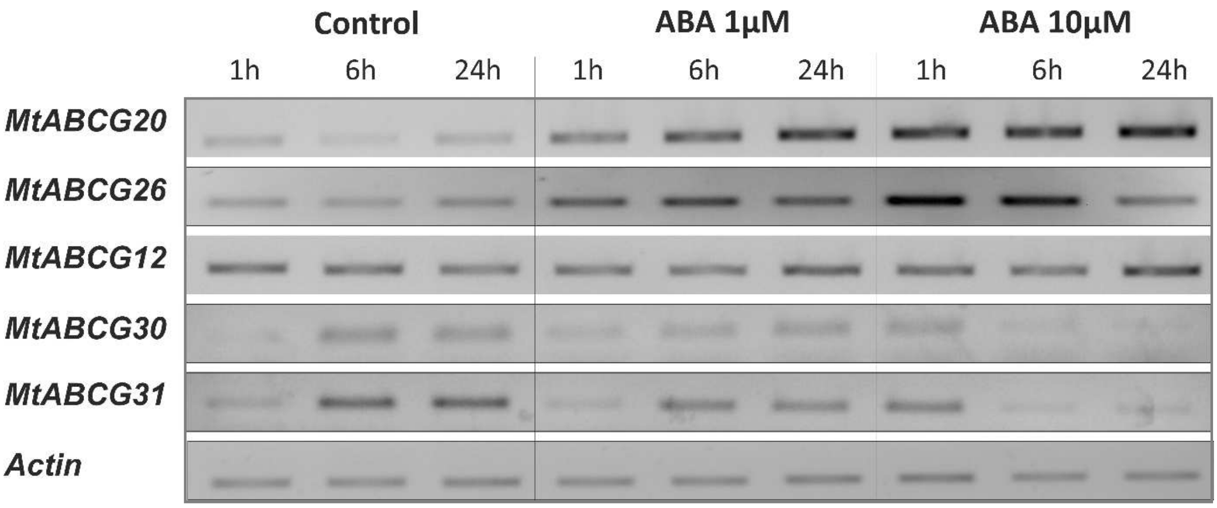

Figure S10. Changes of the selected (clustering with AtABCG25) half-size *MtABCGs* expression levels in roots after exogenous ABA application. Reverse-transcriptase polymerase chain reaction (RT-PCR) analysis of the half-size *MtABCGs* mRNA accumulation in control and ABA (1 µM and 10 µM) treated roots at the indicated time points. The *Actin* gene transcript was used as an internal control.

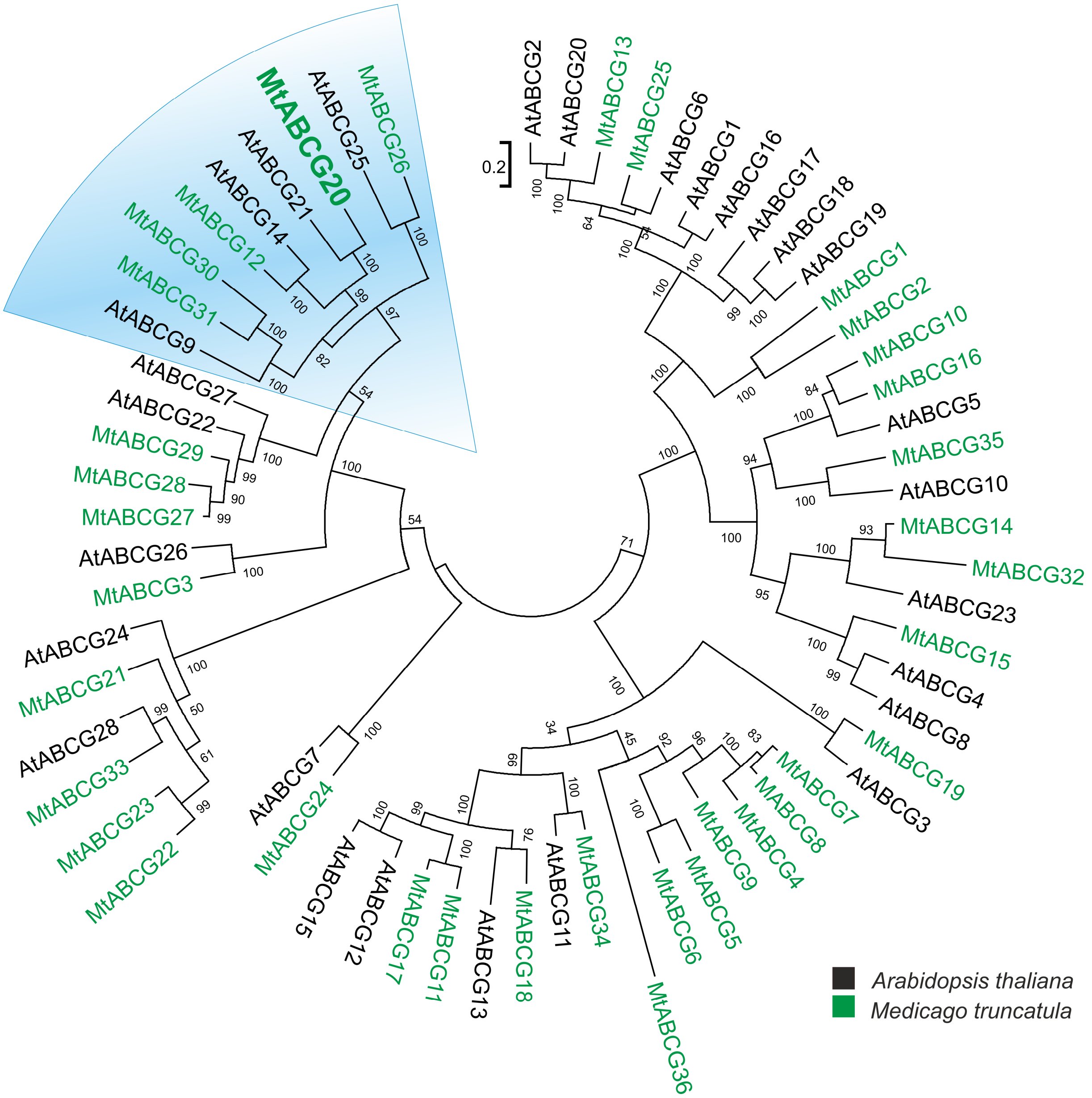

Figure S11. Phylogenetic tree of half-size ABCG proteins from *Arabidopsis thaliana* and *Medicago truncatula*. A maximum likelihood tree (bootstraps: 1000) was conducted using MEGA 6.0 software based on the amino acid sequences after multiple sequence alignment generated with MUSCLE. The cluster with described ABA transporters (AtABCG25 and MtABCG20) is highlighted.

Supporting Tables

Table S1. Accession numbers of *Medicago truncatula* half-size *ABCG* genes (*WBC*).

NAME	NAME*	LOCUS
MtABCG1	*MtWBC1/MtSTR1***	Medtr8g107450
MtABCG2	*MtWBC2/MtSTR2***	Medtr5g030910
MtABCG3	*MtWBC3*	Medtr5g096390
MtABCG4	*MtWBC4*	Medtr4g054020
MtABCG5	*MtWBC5/MtABCG3****	Medtr4g093845
MtABCG6	*MtWBC6*	Medtr4g094090
MtABCG7	*MtWBC7*	Medtr4g094060
MtABCG8	*MtWBC8*	Medtr4g094050
MtABCG9	*MtWBC9*	Medtr4g094010
MtABCG10	*MtWBC10*	Medtr8g093840
MtABCG11	*MtWBC11*	Medtr4g076900
MtABCG12	*MtWBC12*	Medtr5g025470
MtABCG13	*MtWBC13*	Medtr7g100120
MtABCG14	*MtWBC14*	Medtr4g116540
MtABCG15	*MtWBC15*	Medtr1g099570
MtABCG16	*MtWBC16*	Medtr3g096410
MtABCG17	*MtWBC17*	Medtr4g076940
MtABCG18	*MtWBC18*	Medtr4g076970
MtABCG19	*MtWBC19*	Medtr2g095390
MtABCG20	*MtWBC20*	Medtr1g093990
MtABCG21	*MtWBC21*	Medtr1g094660
MtABCG22	*MtWBC22*	Medtr1g063920
MtABCG23	*MtWBC23*	Medtr7g101780
MtABCG24	*MtWBC24*	Medtr1g108340
MtABCG25	*MtWBC25*	Medtr1g115790
MtABCG26	*MtWBC26*	Medtr1g096580
MtABCG27	*MtWBC27*	Medtr1g054935
MtABCG28	*MtWBC28*	Medtr1g054960
MtABCG29	*MtWBC29*	Medtr7g106880
MtABCG30	*MtWBC30*	Medtr8g059150
MtABCG31	*MtWBC31*	Medtr2g079980
MtABCG32	*MtWBC32*	Medtr3g040670
MtABCG33	*MtWBC33*	Medtr4g058000
MtABCG34	*MtWBC34*	Medtr6g066240
MtABCG35	*MtWBC35*	Medtr2g078080
MtABCG36	*MtWBC36*	Medtr4g094080

MtABCG/MtWBC have been identified in *M.truncatula* genome, version Mt4.0v2
http://www.medicagogenome.org/ (Tang et al., 2014)
* MtWBC1-MtWBC25 (Banasiak and Jasinski, 2014);
** (Gutjahr et al., 2012);
*** (Luginbuehl et al., 2017).

Table S2. List of primers used in this study.

A. Primers for Real-Time PCR/ddPCR/semi-quantitative PCR analyses

Genes	Forward primers	Reverse primers
MtABCG20	5'- TCT CAT GGA TGT TAA GCA GG -3'	5'- CTC CCC ACA TAT TAC CAA GC -3'
MtHAI2	5'-CATTGGCGAGGAATAGTTCG-3'	5'-TGTCCAGACACAGTACACG-3'
MtEXP1	5'-GTATAGGAGAGTTGGGTGC-3'	5'-ATAGCTGTACGAGTCTTCC-3'
MtNCED	5'-TTCTATTCAGCTTCCTTCTCG-3'	5'-GTAAAATCTCTACTCACAGACC-3'
MtGPAT5	5'-TTC CTA CCG TGA GAC TAA CC-3'	5'-CTT TCC GAG TAA AGT TAG TGC-3'
abi1-1	5'-CTT CCA TTA TCC GTT GAC C -3'	5'-CAC ACT TAT GTT GTC TTT GC -3'
Mtβ-Actin	5'-GTACTTTCCAGCAGATGTGG-3'	5'-AACCTACAGACATCCAGTGG-3'

B. Promoters activity analyses (gene specific sequences underlined)

DNA fragments	Forward primers	Reverse primers
PrABCG20-GUS	5'- atgaattcGGACGAGTTATTTGTTTAGG-3'	5'-taggatccATCTTAGATATAAGATAAAGTTTTG-3'
PrABCG20-NLS-GFP	5'-tagttggaatgggttcgaaGGACGAGTTATTTGTTTAGG-3'	5'-ttatggagttgggttcgaaCTTAGATATAAGATAAAGTTTTG-3'

C. Primers used for clonning of MtWBC20 cDNA (gene specific sequences underlined)

DNA fragment	Forward primers	Reverse primers
MtABCG20	5'-ACTTTGAGTTTATCCTCTAGCC-3'	5'-GTTAGTAACACTGACACAGG-3'
MtABCG20 AscI/PacI	5'-ggcgcgccTCTTATATCTAAGATGATGC-3'	5'-ttaattaaGGTTGGACCCTAGACACGC-3'
MtABCG20 Gateway	5'-ggggacaagtttgtacaaaaaagcaggcttcCTTATATCTAAGATGATGCC-3'	5'- ggggaccactttgtacaagaaagctgggtcGGCATTTAGGTTGCCC -3'

Supplementary Reference

Banasiak, J., Jasinski, M. (2014). Defence, symbiosis and ABCG transporters. In Plant ABC transporters (Springer: Heidelberg), p.163-184.

Gutjahr, C., Radovanovic, D., Geoffroy, J., Zhang, Q., Siegler, H., Chiapello, M., Casieri, L., An, K., An, G., Guiderdoni, E., et al. (2012). The half-size ABC transporters STR1 and STR2 are indispensable for mycorrhizal arbuscule formation in rice. Plant J 69:906-920.

Jasinski, M., Stukkens, Y., Degand, H., Purnelle, B., Marchand-Brynaert, J., and Boutry, M. (2001). A plant plasma membrane ATP binding cassette-type transporter is involved in antifungal terpenoid secretion. Plant Cell 13:1095-1107.

Luginbuehl, L.H., Menard, G.N., Kurup, S., Van Erp, H., Radhakrishnan, G.V., Breakspear, A., Oldroyd, G.E.D., and Eastmond, P.J. (2017). Fatty acids in arbuscular mycorrhizal fungi are synthesized by the host plant. Science 356:1175-1178.

Tang, H., Krishnakumar, V., Bidwell, S., Rosen, B., Chan, A., Zhou, S., Gentzbittel, L., Childs, K.L., Yandell, M., Gundlach, H., et al. (2014). An improved genome release (version Mt4.0) for the model legume Medicago truncatula. BMC Genomics 15:312.
