# Supplementary material for: MtABCG20 is an ABA exporter influencing root morphology and seed germination of Medicago truncatula
Source: Plant J. 2019 Mar 6;98(3):511–23. doi: 10.1111/tpj.14234 (PMC6850635; doi:10.1111/tpj.14234)
Supplement: Supplementary file 2 — Table S1. Accession numbers of Medicago truncatula half‐size ABCG genes (WBC). Table S2. List of primers used in this study. [file TPJ-98-511-s002.docx]

**SUPPORTING INFORMATION TABLES**

**Supporting Tables**

**Table S1.** Accession numbers of *Medicago truncatula* half-size *ABCG* genes (*WBC*).

| NAME | Name* | Locus |
| --- | --- | --- |
| *MtABCG1* | *MtWBC1/MtSTR1*** | Medtr8g107450 |
| *MtABCG2* | *MtWBC2/MtSTR2*** | Medtr5g030910 |
| *MtABCG3* | *MtWBC3* | Medtr5g096390 |
| *MtABCG4* | *MtWBC4* | Medtr4g054020 |
| *MtABCG5* | *MtWBC5/MtABCG3**** | Medtr4g093845 |
| *MtABCG6* | *MtWBC6* | Medtr4g094090 |
| *MtABCG7* | *MtWBC7* | Medtr4g094060 |
| *MtABCG8* | *MtWBC8* | Medtr4g094050 |
| *MtABCG9* | *MtWBC9* | Medtr4g094010 |
| *MtABCG10* | *MtWBC10* | Medtr8g093840 |
| *MtABCG11* | *MtWBC11* | Medtr4g076900 |
| *MtABCG12* | *MtWBC12* | Medtr5g025470 |
| *MtABCG13* | *MtWBC13* | Medtr7g100120 |
| *MtABCG14* | *MtWBC14* | Medtr4g116540 |
| *MtABCG15* | *MtWBC15* | Medtr1g099570 |
| *MtABCG16* | *MtWBC16* | Medtr3g096410 |
| *MtABCG17* | *MtWBC17* | Medtr4g076940 |
| *MtABCG18* | *MtWBC18* | Medtr4g076970 |
| *MtABCG19* | *MtWBC19* | Medtr2g095390 |
| *MtABCG20* | *MtWBC20* | Medtr1g093990 |
| *MtABCG21* | *MtWBC21* | Medtr1g094660 |
| *MtABCG22* | *MtWBC22* | Medtr1g063920 |
| *MtABCG23* | *MtWBC23* | Medtr7g101780 |
| *MtABCG24* | *MtWBC24* | Medtr1g108340 |
| *MtABCG25* | *MtWBC25* | Medtr1g115790 |
| *MtABCG26* | *MtWBC26* | Medtr1g096580 |
| *MtABCG27* | *MtWBC27* | Medtr1g054935 |
| *MtABCG28* | *MtWBC28* | Medtr1g054960 |
| *MtABCG29* | *MtWBC29* | Medtr7g106880 |
| *MtABCG30* | *MtWBC30* | Medtr8g059150 |
| *MtABCG31* | *MtWBC31* | Medtr2g079980 |
| *MtABCG32* | *MtWBC32* | Medtr3g040670 |
| *MtABCG33* | *MtWBC33* | Medtr4g058000 |
| *MtABCG34* | *MtWBC34* | Medtr6g066240 |
| *MtABCG35* | *MtWBC35* | Medtr2g078080 |
| *MtABCG36* | *MtWBC36* | Medtr4g094080 |

*MtABCG/MtWBC* have been identified in *M.truncatula* genome, version Mt4.0v2 <http://www.medicagogenome.org/> (Tang et al., 2014)

* MtWBC1-MtWBC25 (Banasiak and Jasinski, 2014);

** (Gutjahr et al., 2012);

*** (Luginbuehl et al., 2017).

**Table S2.** List of primers used in this study.

| **A. Primers for Real-Time PCR/ddPCR/semi-quantitative PCR analyses** | |  |
| --- | --- | --- |
| **Genes** | **Forward primers** | **Reverse primers** |
| *MtABCG20* | 5'- TCT CAT GGA TGT TAA GCA GG -3' | 5'- CTC CCC ACA TAT TAC CAA GC -3' |
| *MtHAI2* | 5'-CATTGGCGAGGAATAGTTCG-3' | 5'-TGTCCAGACACAGTACACG-3' |
| *MtEXP1* | 5'-GTATAGGAGAGTTGGGTGC-3' | 5'-ATAGCTGTACGAGTCTTCC-3' |
| *MtNCED* | 5'-TTCTATTCAGCTTCCTTCTCG-3' | 5'-GTAAAATCTCTACTCACAGACC-3' |
| *MtGPAT5* | 5'-TTC CTA CCG TGA GAC TAA CC-3' | 5'-CTT TCC GAG TAA AGT TAG TGC-3' |
| *abi1-1* | 5'-CTT CCA TTA TCC GTT GAC C -3' | 5'-CAC ACT TAT GTT GTC TTT GC -3' |
| *Mtβ-Actin* | 5'-GTACTTTCCAGCAGATGTGG-3' | 5'-AACCTACAGACATCCAGTGG-3' |
|  |  |  |
|  |  |  |
| **B. Promoters activity analyses (gene specific sequences underlined)** | | |
| **DNA fragments** | **Forward primers** | **Reverse primers** |
| *PrABCG20-GUS* | 5'- atgaattcGGACGAGTTATTTGTTTAGG-3' | 5'-taggatccATCTTAGATATAAGATAAAGTTTTG-3' |
| *PrABCG20-NLS-GFP* | 5'-tagttggaatgggttcgaaGGACGAGTTATTTGTTTAGG-3' | 5'-ttatggagttgggttcgaaCTTAGATATAAGATAAAGTTTTG-3' |
|  |  |  |
|  |  |  |
| **C. Primers used for clonning of MtWBC20 cDNA (gene specific sequences underlined)** | | |
| **DNA fragment** | **Forward primers** | **Reverse primers** |
| *MtABCG20* | 5'-ACTTTGAGTTTATCCTCTAGCC-3' | 5'-GTTAGTAACACTGACACAGG-3' |
| *MtABCG20* AscI/PacI | 5'-ggcgcgccTCTTATATCTAAGATGATGC-3' | 5'-ttaattaaGGTTGGACCCTAGACACGC-3' |
| *MtABCG20* Gateway | 5'-ggggacaagtttgtacaaaaaagcaggcttcCTTATATCTAAGATGATGCC-3' | 5'- ggggaccactttgtacaagaaagctgggtcGGCATTTAGGTTGCCC -3' |
