# Supplementary material for: MtABCG20 is an ABA exporter influencing root morphology and seed germination of Medicago truncatula
Source: Plant J. 2019 Mar 6;98(3):511–23. doi: 10.1111/tpj.14234 (PMC6850635; doi:10.1111/tpj.14234)
Supplement: Supplementary file 3 [file TPJ-98-511-s003.docx]

**SUPPORTING INFORMATION REFERENCES**

Banasiak, J., Jasinski, M. (2014). Defence, symbiosis and ABCG transporters. In Plant ABC transporters (Springer: Heidelberg), p.163-184.

Gutjahr, C., Radovanovic, D., Geoffroy, J., Zhang, Q., Siegler, H., Chiapello, M., Casieri, L., An, K., An, G., Guiderdoni, E., et al. (2012). The half-size ABC transporters STR1 and STR2 are indispensable for mycorrhizal arbuscule formation in rice. Plant J 69:906-920.

Jasinski, M., Stukkens, Y., Degand, H., Purnelle, B., Marchand-Brynaert, J., and Boutry, M. (2001). A plant plasma membrane ATP binding cassette-type transporter is involved in antifungal terpenoid secretion. Plant Cell 13:1095-1107.

Luginbuehl, L.H., Menard, G.N., Kurup, S., Van Erp, H., Radhakrishnan, G.V., Breakspear, A., Oldroyd, G.E.D., and Eastmond, P.J. (2017). Fatty acids in arbuscular mycorrhizal fungi are synthesized by the host plant. Science 356:1175-1178.

Tang, H., Krishnakumar, V., Bidwell, S., Rosen, B., Chan, A., Zhou, S., Gentzbittel, L., Childs, K.L., Yandell, M., Gundlach, H., et al. (2014). An improved genome release (version Mt4.0) for the model legume Medicago truncatula. BMC Genomics 15:312.

{Tang, 2014 #61}
