# Supplementary material for: MtABCG20 is an ABA exporter influencing root morphology and seed germination of Medicago truncatula
Source: Plant J. 2019 Mar 6;98(3):511–23. doi: 10.1111/tpj.14234 (PMC6850635; doi:10.1111/tpj.14234)
Supplement: Supplementary file 4 [file TPJ-98-511-s004.docx]

**SUPPORTING INFORMATION LEGENDS**

**Figure S1.** Promoter activity analyses of *MtABCG20* in transgenic *M. truncatula* roots. (A) Control, untreated transgenic roots. (B) Transgenic roots treated by 10 µM ABA.

**Figure S2.** Promoter activity analysis of *MtABCG20* in transgenic *M. truncatula* nodule.

**Figure S3.** Phenotypic characterization of *mtabcg20* mutants. (A) Average lateral root number per plant in WT and *mtabcg20* plants. All plants were grown for four weeks on ½ MS medium. Data represent the mean ± SD of N=5, n=5. (B) Average nodule number per plant in WT and *mtabcg20* plants. 3-day-old seedlings, were inoculated with *S. meliloti* and grown on modified Fahraeus (-N) medium. At 21 days post-inoculation (dpi), nodule numbers were counted. The data represent the mean ± SD of N=5, n=5.

**Figure S4.** Expression of MtABCG20 in *N. tabacum* BY-2 cells. (A) Western blot analysis of the crude membranes (30 μg), obtained from BY2 transformed with empty vector (EV) and BY2 MtABCG20-overexpressing lines. Microsomal fractions were isolated as previously described (Jasinski et al., 2001) from 300 mg of BY2 cells. The proteins were separated by SDS-PAGE and transferred to a polyvinylidene fluoride membrane (Millipore) by electroblotting (semi-dry; apparatus; Bio-Rad). The membrane was incubated with a primary Anti-GFP from mouse (Roche) diluted 1/1000 and the secondary alkaline phosphatase-conjugated goat anti-mouse IgG (Abcam) diluted 1/15000. (B) Coomassie blue-stained gel with crude membrane fraction as a loading control. M - Perfect^TM^ Tricolor Protein Ladder (EURx).

**Figure S5.** Plasma membrane localization of MtABCG20 in BY2 cells. (A) Non-plasmolysed BY2 cell expressing the fusion protein GFP-MtABCG20. GFP signal was localized on the surface of cells (B) Plasmolysed (5% NaCl for 10 min) BY-2 cells expressing the fusion protein GFP-MtABCG20. GFP signal was distributed on a plasma membranes and Hechtian strands. (C) Control BY2 cell expressing free cytoplasmic GFP. Bars = 15 µm.

**Figure S6.** ABA transport assay in BY2 cells. ABA efflux from BY2 control (EV) and *MtABCG20*-overexpressing cell lines, conducted at 18°C and monitored by HPLC/MS. The 100% value represents the quantity of cell-associated ABA, defined as the ratio of the single-ion chromatogram peak area to the internal standard, at the time 0 (T0). Values represent the mean of three experiments ± SD. Significant differences between control and overexpressing lines determined by Student’s t-test are indicated: *P < 0.05, **P < 0.01.

**Figure S7.** Experimental scheme of ABA application onto Medicago embryo**.**

**Figure S8.** The second biological replicate of real-time PCR expression analyses of *MtHAI2* and *MtEXP1* in embryo axes derived from WT and *mtwbc20* dissected embryos. Embryos were untreated or treated with ABA applied onto the hypocotyl-radicle region. Transcript levels were normalized to the *Actin* gene. The data represent the mean ± SD of three technical repeats. Significant differences from the WT plants determined by Student’s t-test are indicated: *P<0.05, **P < 0.01.

**Figure S9.** Two biological replicates of real-time PCR expression analyses of *MtHAI2* in cotyledons derived from WT and *mtwbc20* dissected embryos. Embryos were untreated or treated with ABA applied to the hypocotyl-radicle region. Transcript levels were normalized to the *Actin* gene. The data represent the mean ± SD of three technical repeats. Significant differences from the WT plants determined by Student’s t-test are indicated: *P<0.05, ***P<0.001.

**Figure S10.** Changes of the selected (clustering with AtABCG25) half-size *MtABCGs* expression levels in roots after exogenous ABA application. Reverse-transcriptase polymerase chain reaction (RT-PCR) analysis of the half-size *MtABCGs* mRNA accumulation in control and ABA (1 µM and 10 µM) treated roots at the indicated time points. The *Actin* gene transcript was used as an internal control.

**Figure S11.** Phylogenetic tree of half-size ABCG proteins from *Arabidopsis thaliana* and *Medicago truncatula*. A maximum likelihood tree (bootstraps: 1000) was conducted using MEGA 6.0 software based on the amino acid sequences after multiple sequence alignment generated with MUSCLE. The cluster with described ABA transporters (AtABCG25 and MtABCG20) is highlighted.

**Table S1.** Accession numbers of *Medicago truncatula* half-size ABCG genes (WBC).

**Table S2.** List of primers used in this study.

{Tang, 2014 #61}
